# A Functional Near-Infrared Spectroscopy Examination of the Neural Correlates of Cognitive Shifting in Dimensional Change Card Sort Task

**DOI:** 10.3389/fnhum.2020.561223

**Published:** 2021-01-21

**Authors:** Hui Li, Dandan Wu, Jinfeng Yang, Sha Xie, Jiutong Luo, Chunqi Chang

**Affiliations:** ^1^School of Education, Macquarie University, Sydney, NSW, Australia; ^2^School of Biomedical Engineering, Shenzhen University, Shenzhen, China; ^3^Normal College, Shenzhen University, Shenzhen, China; ^4^Faculty of Education, Beijing Normal University, Beijing, China

**Keywords:** dimensional change card sort task, functional near-infrared spectroscopy, neural correlates, cognitive shifting, developmental pattern

## Abstract

This study aims to examine the neural correlates of cognitive shifting during the *Dimensional Change Card Sort Task (DCCS)* task with functional near-infrared spectroscopy. Altogether 49 children completed the *DCCS* tasks, and 25 children (M_age_ = 68.66, SD = 5.3) passing all items were classified into the Switch group. Twenty children (M_*age*_ = 62.05, SD = 8.13) committing more than one perseverative errors were grouped into the Perseverate group. The Switch group had Brodmann Area (BA) 9 and 10 activated in the pre-switch period and BA 6, 9, 10, 40, and 44 in the post-switch period. In contrast, the Perseverate group had BA 9 and 10 activated in the pre-switch period and BA 8, 9, 10 in the post-switch period. The general linear model results afford strong support to the “V-shape curve” hypothesis by identifying a significant decrease–increase cycle in BA 9 and 44, the neural correlations of cognitive shifting.

## Introduction

Cognitive shifting is a kind of ability to switch between different mental tasks flexibly and is widely regarded as a remarkable milestone in early cognitive development (Moriguchi and Hiraki, 2009, 2011, 2014; Buss and Spencer, 2014; Perone et al., 2015, 2019). In the past decade, many neuroimaging studies have examined cognitive shifting using the *Dimensional Change Card Sort* (DCCS) (see Moriguchi, 2017, for a review). In the DCCS task, children are provided with test cards with two dimensions (color and shape) and are asked to sort the cards into trays with target cards (e.g., a blue rabbit and a red boat). During the pre-switch period, children are asked to sort cards according to one dimension (e.g., shape) for several trials. Children are then invited to sort the cards according to the other dimension (e.g., color) for several trials during the post-switch periods. Many studies (i.e., Moriguchi and Hiraki, 2009, 2011, 2014) have recently examined the DCCS task using functional near-infrared spectroscopy (fNIRS) and included all the activated brain areas as the “neural correlates” of cognitive shifting. This is problematic, as some brain areas are generally involved in the background and supportive systems; only a few areas are specifically responsible for the initiating, controlling, and monitoring cognitive shifting needed in this task. To fill this gap, this study will develop a new indicator to judge the occurrence of cognitive shifting in the DCCS task and to evaluate its applicability and effectiveness in the fNIRS study.

## Dimensional Change Card Sort Task and Neurocognitive Development

The DCCS task asks children to sort objects by one dimension (e.g., shape) during the pre-switch period before switching to the other dimension (e.g., color) in the post-switch period. Buss and Spencer (2014) suggest that DCCS is ideal for studying early neurocognitive development for three reasons. First, it requires inhibition to suppress the irrelevant dimension's processing and working memory to maintain representations of the relevant task rules and cognitive shifting to update these processes after the rule-switch. Second, DCCS can reveal rapid and dramatic changes in early cognitive development: most 3-year-olds fail the task and persevere on the first set of rules, whereas 5-year-olds have little trouble switching rules. Third, DCCS provides an ideal foundation for developing theories that integrate behavior and brain studies. Also, previous studies on the brain–behavior connection have revealed changes in a network of brain areas associated with the cognitive-shifting that emerges after age 3 years and becomes more refined in adulthood (Moriguchi and Hiraki, 2009, 2011; Morton et al., 2009). Thus, the DCCS task can provide insights into the wide spectrum of executive function (EF) processes and the associated neural changes in early childhood (Buss and Spencer, 2014).

Different theoretical frameworks have been proposed to explain the neurocognitive development of DCCS in the past decade. The Cognitive Complexity and Control-revised theory suggests that children need to formulate and use a higher-order rule for selecting rules (color or shape) when switching dimension, which is challenging for young children (Zelazo et al., 2003). The Attentional Inertia Theory postulates that children perseverate in the post-switch period because of the inability to inhibit attention to the first dimension and to shift attention to the second dimension (Kirkham et al., 2003). Another EF-related developmental theory is the representational redescription that children's failure in switching lies in their difficulty to represent a single stimulus as represented from different perspectives (Perner and Lang, 2002; Kloo and Perner, 2005). Also, finally, from the working memory perspective, children have to actively maintain information in working memory, which allows the maintained rules to outcompete latent representations corresponding to the pre-switch rules (Morton and Munakata, 2002).

Apart from these developmental theories of EF, computational models have also been proposed to interpret the neurocognitive development of DCCS (Marcovitch and Zelazo, 2000; van Bers et al., 2011; Buss and Spencer, 2018). For instance, Buss and Spencer (2014) proposed the Dynamic Neural Field (DNF) model: when a child is instructed to sort by one dimension during the pre-switch period, for example, sorting by shape, he or she will activate the shape node, which in turn boosts the shape working memory field and leads to the accumulation of strong memory traces. However, when the post-switch period begins, the child is instructed to sort by color, which will selectively activate the color node in the frontal system and, accordingly, will boost the color working memory field in the posterior system. However, if the connection weights between the frontal and posterior systems are relatively weak thus cannot overcome the strong memory traces in the posterior system for sorting by shape, the child will perseverate on the pre-switch dimension (sorting by shape), like 3-year-old children. Thus, the strength of connections between the frontal and posterior systems in the DNF model is a key source of developmental change in children's performance in the DCCS task (Perone et al., 2015, 2019). This DNF model implies that the substantial engagement of both frontal and posterior systems might be critical to completing cognitive shifting in the DCCS task. In particular, as the major part of the pre-frontal cortex, Brodmann Area (BA) 9 is substantially involved in neurocognitive processing such as attention, working memory, conflict monitoring, and problem-solving. Therefore, we first hypothesize that BA 9 might be one of the neural correlates of cognitive shifting in the DCCS task.

## Neural Correlates of Dimensional Change Card Sort Task

Moriguchi and his team have extensively explored the neural correlates of the DCCS task by using fNIRS to measure the concentration of oxygenated hemoglobin (HbO). Moriguchi and Hiraki (2009), for the first time, found that 5-year-olds and adult participants flawlessly performed the task and showed significant activation in the bilateral inferior pre-frontal cortex during the pre-switch and post-switch periods. However, some 3-year-olds committed perseverative errors during the post-switch period. This fNIRS finding indicated that the right inferior pre-frontal cortex might be the neural basis of the DCCS task.

Later, Moriguchi and Hiraki (2011) examined the developmental changes in pre-frontal cortex activation with a 2-year longitudinal study and found that: (1) children who performed better in the DCCS task showed significant activation of the right inferior pre-frontal cortex at Time 1 (age 3 years) and significant activation of the bilateral inferior pre-frontal cortex at Time 2 (age 4 years); (2) children who performed poorer exhibited no significant activation of the inferior pre-frontal cortex at Time 1 (age 3 years) but significant activation of left inferior pre-frontal cortex at Time 2 (age 4 years). Accordingly, they concluded that pre-frontal cortex activation might play an essential role in successful shifting during the DCCS task, and there would be individual differences in the development of such activation patterns.

Furthermore, Moriguchi and Hiraki (2014) examined how young children and adult participants activated inferior pre-frontal regions when given two versions of the DCCS (the standard vs. the advanced) using fNIRS. In the advanced DCCS, children needed to switch flexibly between two incompatible rules within the same set. For example, half of the test cards had a border around them, and children were asked to sort according to one rule if the card had a border and according to another rule if the card had no border. Their study showed significant differences in activations between the regions during the advanced version but not during the standard version. In contrast, the adults exhibited similar bilateral inferior pre-frontal activations during the two versions of DCCS. These results indicated that young children might be developing their neural correlates for the DCCS task and had to activate different, inferior pre-frontal areas to meet the different demands of cognitive shifting.

Based on the previous studies, Moriguchi et al. (2015) conducted a training study and found that behavioral changes during the DCCS task might also be related to the changes in the activation of the left pre-frontal cortex that are responsible for EF. Recently, Moriguchi and Shinohara (2018) examined the pre-frontal activations using the Minnesota version of the DCCS, which asked children to switch from color to shape to color and found the association between gene polymorphism and pre-frontal activations in young children. All these neuroimaging studies have indicated the involvement of pre-frontal areas in cognitive shifting. However, based on the DNF model (Buss and Spencer, 2014), both the pre-frontal and posterior cortices should be involved in the cognitive shifting of the DCCS task. Therefore, this study will explore the possible neural correlates located in the pre-frontal and posterior cortex.

## This Study

Most of the fNIRS studies (Moriguchi and Hiraki, 2009, 2011, 2014) on the DCCS task simply analyzed the changes in HbO between the task and baseline conditions and, accordingly, have included all the activated brain areas involved in the DCCS task. However, the specific neural correlates responsible for cognitive shifting could not be identified using this research paradigm. This is because some brain areas are generally involved in completing the DCCS task and playing a supportive role. In contrast, some specific brain areas are exclusively responsible for initiating, controlling, and monitoring the cognitive shifting needed in this task. Therefore, a more direct and critical indicator to demonstrate cognitive shifting in the DCCS task should be developed and used in this type of study.

Habituation is one of the fundamental mechanisms underlying human being's cognition and behavior: when the same stimulus is repeated over and over, there will be reduced response from the same neural correlates and, accordingly, a decrease in HbO in the blood (Purves et al., 2018). However, as soon as there are some changes in the stimulus or rules or simply a new task, the habituated brain areas will have an increased response and, accordingly, a significant increase in HbO in the blood, which is called dishabituation (Purves et al., 2018). The habituation and novelty detection (HaND) paradigm (Lloyd-Fox et al., 2019) has been widely used to explore the development of discriminatory neural responses associated with attention, learning, and memory mechanisms in the early years. Recently, several recent fNIRS studies have shown that repeated exposure to identical stimuli could produce neural habituation in very young infants (Benavides-Varela et al., 2011; Bouchon et al., 2015) and recovery of response to novelty (e.g., a speaker change) (Benavides-Varela et al., 2011).

The DCCS task has a similar HaND paradigm: children are asked to sort the cards using two different rules, for example, sorting by color in the pre-switch period (Sub-task 1), sorting by shape in the post-switch period (Sub-task 2). Similar to the HaND paradigm (Lloyd-Fox et al., 2019), children would demonstrate habituation when repeating Sub-task 1. When starting Sub-task 2, which is also a new task to them, children would also have to activate all the necessary neural correlates. Therefore, there should be an observed dishabituation in the correlated brain areas. Based on the DNF model (Perone et al., 2015, 2019), we assume that the children fail to complete Sub-task 2 correctly because their executive function (BA 9, the pre-frontal system) fails to alert the posterior system (i.e., BA 40, BA 44) of the changes in the sorting rules. Therefore, all the neural correlates of the cognitive shifting in this DCCS should have seen habituation and dishabituation in the pre- and post-switch periods, respectively. Consequently, there should be a significant decrease in HbO (habituation) in the pre-switch period and a significant increase in HbO (dishabituation) in the post-switch period. Taking time as the independent variable “X” and the HbO value of each second (time-sampling) as the dependent variable “Y” in the regression analysis, and “ε” as the error term, there should be a V-shaped curve to reflect the significant decrease and increase in HbO for the pre-switch and post-switch periods. This V-shaped curve could be verified by using a pair of general linear model (GLM) analyses to estimate the changes in HbO (Δ*HbO*) for each channel based on time, using the same regression formula:

$$\begin{array}{l} Y_{\Delta HbO}=aX_{time}+b\;+\varepsilon \end{array}$$

In this formula, a significant decrease in HbO (“Y_Δ*HbO*_”) should be observed during the pre-switch period. In contrast, a significant increase in HbO (“Y_Δ*HbO*_”) should also be observed in the post-switch period. If *a*_*pre*−*switch*_ is negative (-*a)*, whereas *a*_*post*−*switch*_ is positive (+*a)*, and both models are significant, a perfect V-shaped curve could be verified, and the corresponding channel could be confirmed as the neural correlate of “cognitive shifting.” Accordingly, we hypothesize that this V-shaped curve is a more direct indicator of cognitive shifting and will test it in this study. In particular, the following questions guided this study.

1. Will the preschoolers pass all the DCCS testing items correctly?
2. What are the differences in the neural networks of the DCCS task between the Switch and Perseverate children?
3. What are the neural correlates of cognitive shifting indicated by the fNIRS evidence?

In particular, we tested the following hypotheses in this study:

H1: BA 9 is one of the specific neural correlates of the cognitive shifting in the DCCS tasks;
H2: A V-shaped curve should be found in the GLM of changes in HbO in the brain areas responsible for the cognitive shifting.

## Methods

### Participants

Altogether 56 right-handed preschoolers (aged between 49 and 75 months, *M* = 66.15 months, *SD* = 7.2 months, 24 girls) were invited to participate in this study. All the parents of these children provided written consent and were informed of the study's purpose and the safety of the fNIRS experiments, which had been reviewed and approved by the University Ethics Committee. The participants were recruited from the same kindergarten in China based on the following selection criteria: (1) right-handed; (2) normal intelligence; (3) normal vision; and (4) capable of completing the experiment. Seven participating children failed to complete the experiments and were thus excluded. Altogether, 49 children (N_4−yr_ = 10; N_5−yr_ = 22; N_6−yr_ = 17) completed the experiments.

### Measures

#### DCCS Task

**Figure 1:**
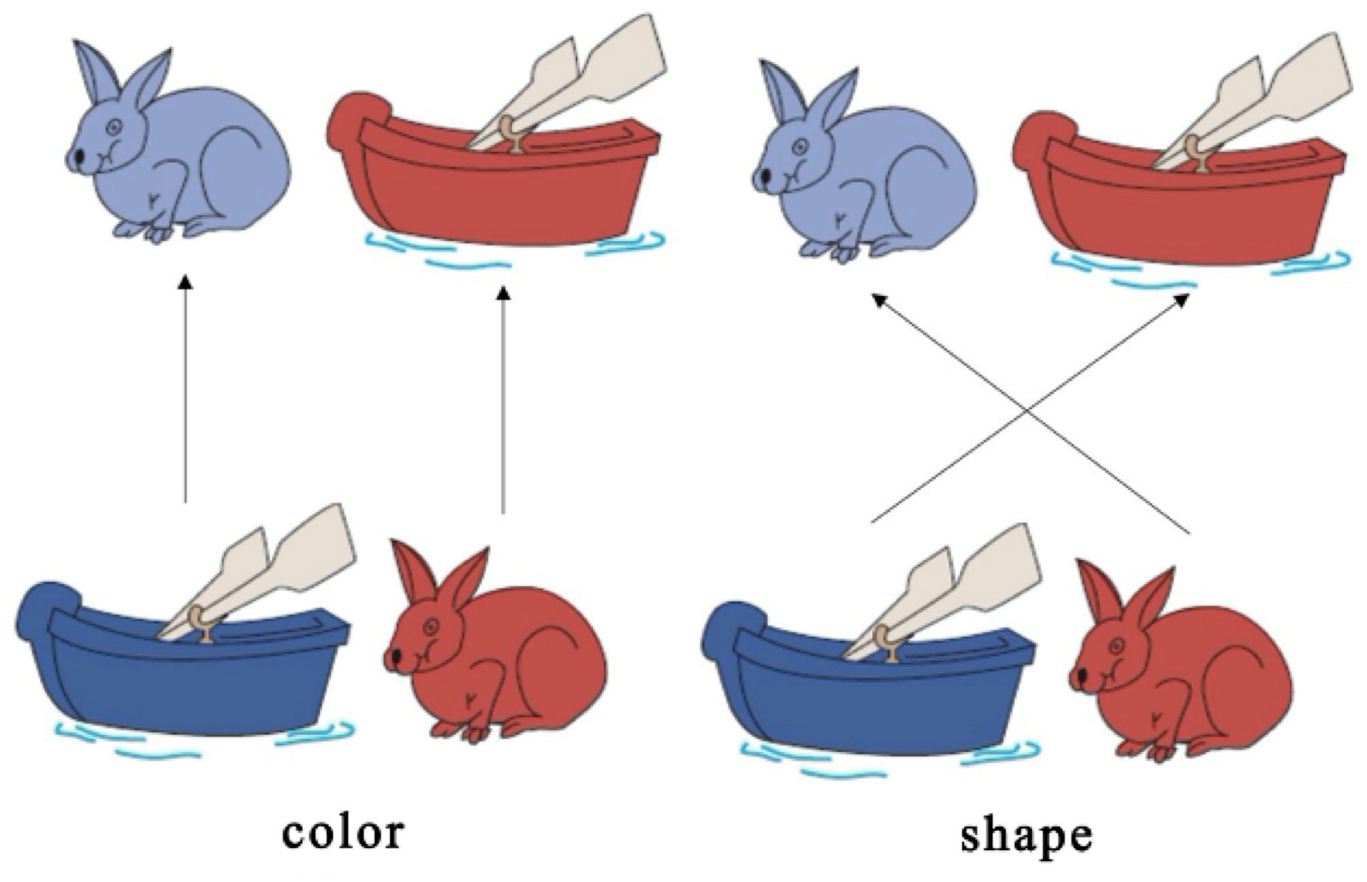
Dimensional Change Card Sort (DCCS) task Zelazo (2006).

**Figure 2:**
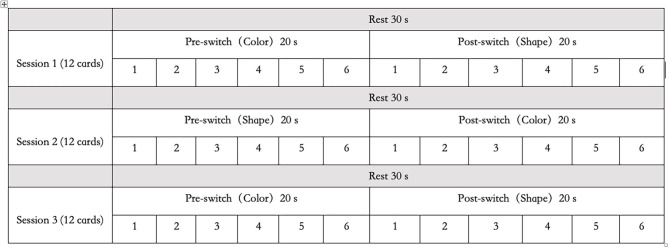
Habituation–dishabituation paradigm.

A set of white paper cards (3.5 × 7.0 cm) was used as the study's stimuli. This set included two target cards (one red boat and one blue rabbit) and 36 test cards (18 red rabbits and 18 blue boats). All the red boat and blue rabbit figures were color printed on the paper card, in the same size. They could be easily distinguished either by shape (boat *vs*. rabbit) or by color (red *vs*. blue). The test cards could match the two target cards either by shape (boat or rabbit) or by color (red or blue). There were no test cards that were identical to the target cards. In this study, the two target cards (red boat and blue rabbit) were placed on the corresponding trays and were used for the three sessions, as shown in Figure 1. Each session consisted of a 30-s rest period (control 1), a 20-s pre-switch period (six cards/trials), and a 20-s post-switch period (six cards/trials), as shown in Figure 2. The participants were given no instructions during the rest periods and were instructed to sort tasks with the corresponding rules during the pre-switch and post-switch periods. During the pre-switch period, the participants were asked to sort the cards according to the first rule (by color). During the post-switch period, the participants were asked to sort the cards according to the second rule (by shape). The rules changed in the three sessions: Session 1: color → shape; Session 2: shape → color; and Session 3: color → shape. This fixed-order was applied to all the participants, resulting in more color-to-shape switches in this study.

The task arrangement in this study has been developed from the HaND paradigm (Lloyd-Fox et al., 2019). It could be named the “habituation–dishabituation paradigm of DCCS task,” which differs from the task arrangement in the previous study (Moriguchi and Lertladaluck, 2020). First, this arrangement asked children to follow three rule orders during the three sessions to control learning effects. Second, each pre-switch and post-switch period was fixed to 20 s each, and each rest period was 30 s. Each participant underwent a training session and three sessions of the DCCS task. The training included six trials and allowed correction when children misunderstood the rules. Last but not least, this arrangement has maximized the chances of habituation and dishabituation in the participating children, as the first period of the following session followed the same sorting rule of the second period of the previous session: Session 1: color → shape; Session 2: shape → color; and Session 3: color → shape. In this way, the children tended to be habituated when they knew the second round of sorting cards should follow the same rule. In another word, this “habituation–dishabituation paradigm of DCCS task” has helped to trigger the occurrence of habituation and dishabituation, thus contributed greatly to the development of the “V-shape” curve theory in this study.

During the training and the experimental sessions, the participants were given detailed instructions regarding the rules and were asked to sort the cards according to different rules. For example, in Session 2, during the pre-switch phase, the children were instructed to follow the first rule: “This is a shape game. All of the boats go here, and all of the rabbits go there.” During the post-switch phase, the children were instructed to follow the second rule: “This is a color game. All of the red cards go here, and all of the blue cards go there.” There was no time limitation in the training session, and children were allowed to complete the task at their own pace. In the pre- and post-switch periods of the testing sessions, time was blocked to 20 s for each period. In the pilot and preparation stages, we found that most children could complete the testing items within 20 s. The correct responses for each participant were recorded, and the percentages were analyzed.

**Figure 3:**
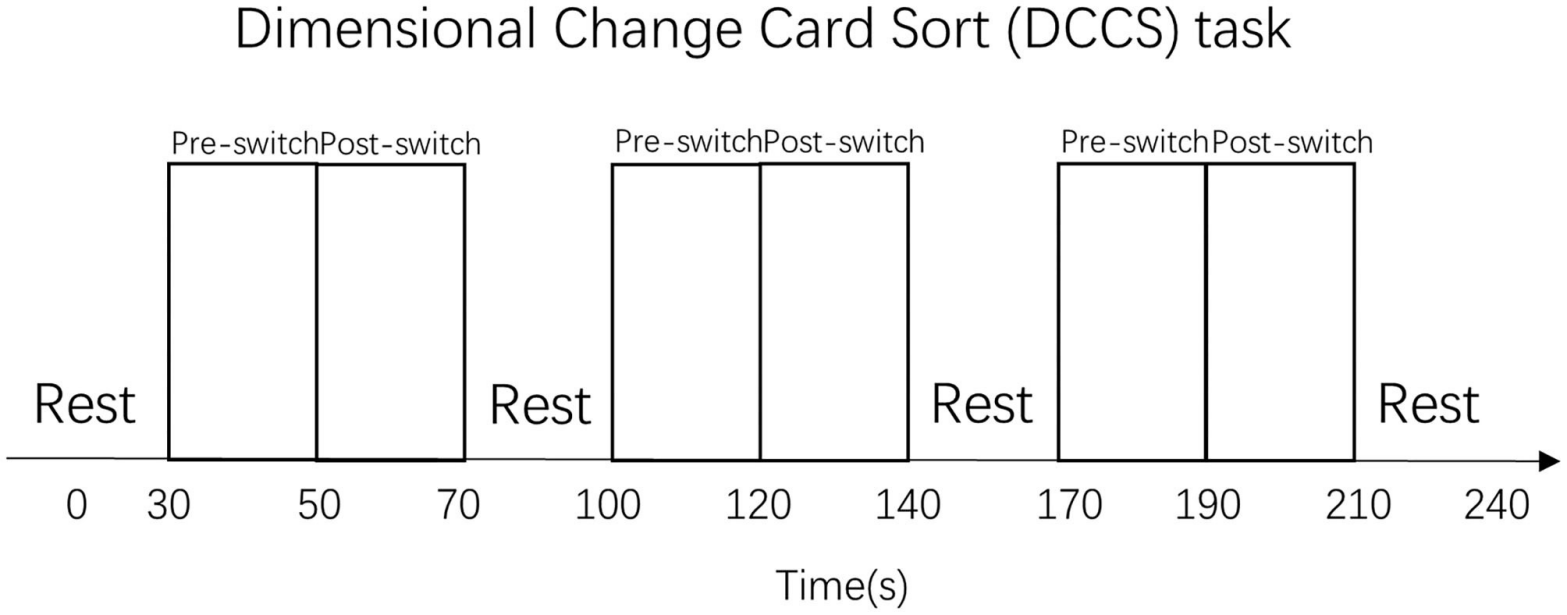
Dimensional Change Card Sort (DCCS) task paradigm. That was a periodic block design involving rest (30 s) and task (40 s) blocks. All the participants performed three sets of the DCCS task and rest blocks.

Different from the standard DCCS task that “a child needs to sort at least five out of six post-switch trials correctly to pass” (Zelazo, 2006), the present study has changed the rule slightly as follows: a child needs to sort all the pre-switch trials (six out of six trials) and all the post-switch trials (six out of six trials) correctly to pass the three sessions. This means that all the children in the pass group have completed all the needed cognitive shifting for all the tasks, thus could be labeled as the “switch” group (van Bers et al., 2011). The DCCS task paradigm is shown in Figure 3.

#### Functional Near-Infrared Spectroscopy Examination

In this study, we used the fNIRS technique to monitor brain activity by measuring changes in hemoglobin concentrations in the outer cortex. Compared with an electroencephalogram, fNIRS offers a more spatially resolved image of activation. In addition, compared with functional magnetic resonance imaging, fNIRS is portable, silent, has a high temporal resolution, and can measure both oxy- and de-oxyhemoglobin chromophores (Lloyd-Fox et al., 2010). Thus, it is particularly suitable for neuroimaging studies on infants and children, especially when doing the DCCS task. In this study, a multiple-channel fNIRS system (Oxymon Mk III, Artinis, The Netherlands) was used to simultaneously measure the concentration changes of HbO, deoxygenated hemoglobin, and total hemoglobin in the participants. Two wavelengths in the near-infrared range (i.e., 760 and 850 nm) were used to measure the changes in optical density and then converted into changes in the concentration of HbO and deoxygenated hemoglobin using the modified Beer–Lambert law.

### Procedure

#### Cap Placement

In this study, we used the child caps accompanied by the NIRS instrument (Oxymon Mk III, Artinis, The Netherlands), which are a highly stretchable soft headwear covering the entire head. The caps have digitized the optode positions to illustrate which parts of the brain are under investigation. First, we took general head measurements to decide the NIRS cap's size for each participant. Both the S and XS size of NIRS caps were used in this study to fit the head size of Chinese preschoolers. Second, the experienced NIRS technician conducted cap placement, hair manipulation and tossing, and optodes installation (based on the 10/20 system). An additional colorful hairband was used to keep the cap in place and to prevent sliding. Third, this process usually took up to a half hour, so the participant was engaged in storybook reading with an experienced preschool teacher.

#### Channel Matching

**Figure 4:**
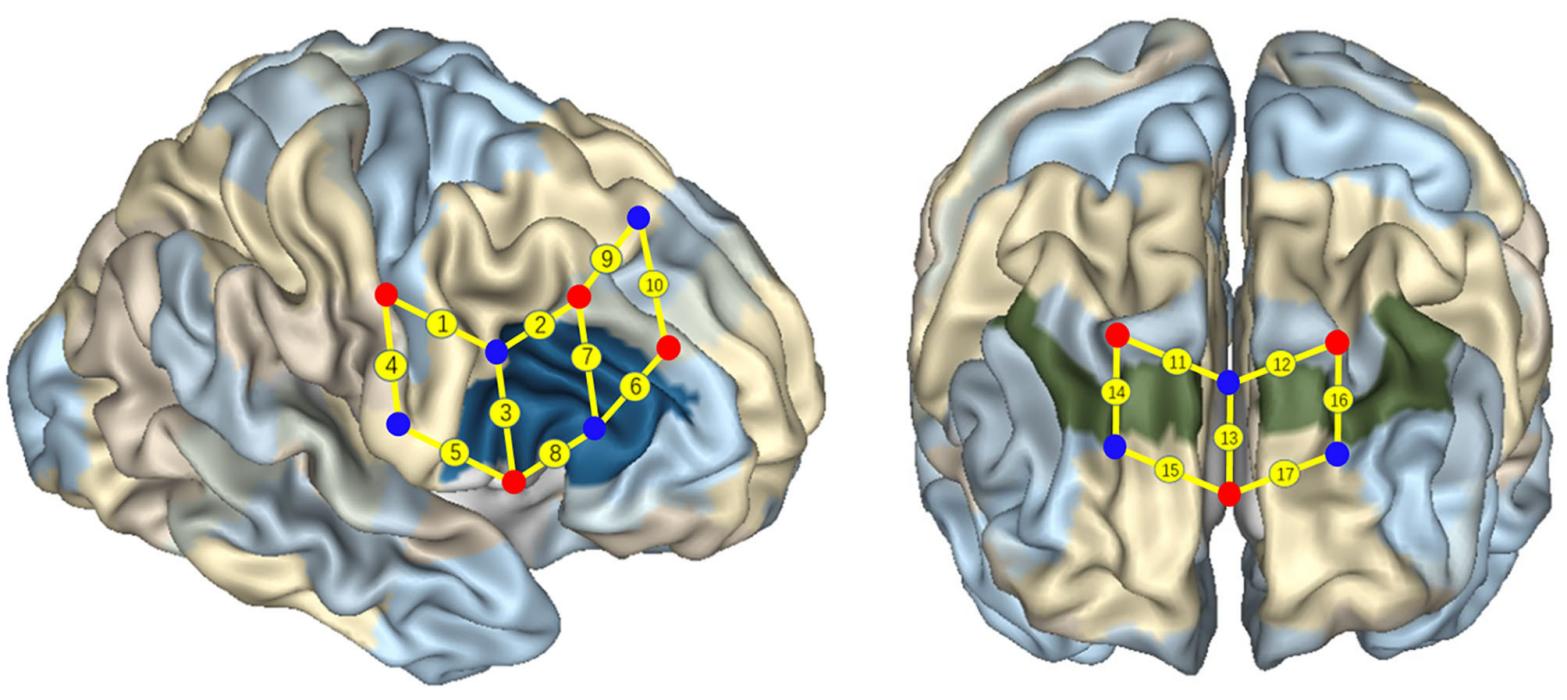
Localization of regions of interest. Numbers on small spheres on the brain map indicate the 17 channels. Channel localization was based on the upper central probe, which was anchored at Fz according to the international 10–20 system and was located at the midpoint between channels numbers 11 and 12. Channels 1 and 9 were located in BA 6, channel 10 was located in BA 8, channels 11, 12, 14, and 16 were located in BA 9, channels 13, 15, and 17 were located in BA 10, channel 4 was located in BA 40, and channels 2, 3, 5, 6, 7, and 8 were located in the right IFC (BA 44).

The 17 channels were located following the international 10/20 system for electroencephalogram, with a 2.5-cm distance between each paired emitters and detectors. The region of interest was located at BAs 6/8/9/10/40/44 (Figure 4), as previous studies have shown that these areas might be activated during cognitive shifting tasks in preschool children. In particular, channels 1 and 9 were located in BA 6, channels 13, 15, and 17 were located in BA 10, channel 10 was located in BA 8, channels 11, 12, 14, and 16 were located in BA 9, channel 4 was located in BA 40 (temporoparietal junction), and channels 2, 3, 5, 6, 7, and 8 were located in BA 44 (inferior frontal cortex). A participant-specific differential path-length factor constant was calculated based on each participant's age (Duncan et al., 1996). Also, the sampling rate was set at 50 Hz for data acquisition.

#### Data Processing

Some of Homer2 NIRS processing package functions (Huppert et al., 2009) were applied to perform the data processing based on MATLAB (Mathworks, MA USA). All incorrect trials were discarded from the analysis. The raw optical intensity data series were converted into changes in optical density (OD) for every participant. Channels with a very low optical intensity were discarded from the analysis using the function enPruneChannels. The discrete wavelet transform was applied to every channel data series to remove motion artifacts, whereas the tuning parameter (α) of wavelet filtering was set to 0.1. Then, the motion detection algorithm hmrMotionArtifact was applied to the OD data to identify motion artifacts; trials that still have motion artifacts were deleted. To reduce very slow drifts and high-frequency noise, a band-pass filter (third-order Butterworth filter) with cutoff frequencies of 0.01–0.3 Hz (Delpy et al., 1988) was then applied to the data. Using the modified Beer–Lambert Law (Delpy et al., 1988), the OD data were converted into concentration changes. Finally, to recover the mean hemodynamic response, all remaining blocks under the same condition were block-averaged.

#### Data Analysis

For each participant, fNIRS data collected during the three sessions of pre-switch periods (20 s), post-switch periods (20 s), and two rest periods (10 s each) before and after the task were analyzed. During each session, the cognitive shift starts at 20 s after each session onset, and the task duration is 40 s after the onset of each session, which are thus divided into the pre-switch period (−20–0 s) and post-switch period (0–20 s). First, two sets of *t*-tests were conducted to explore the between-group and within-group differences in HbO changes in the 17 channels. This analysis will help to understand the significant changes in activation of the region of interest areas (channel) between the different periods (pre-switch vs. post-switch) and the different groups (Switch vs. Perseverate groups). Second, a set of GLM analyses was conducted to model the change in HbO for each channel. This is because GLM has been extensively used in fNIRS studies to estimate the participant, channel, and task-specific evoked hemodynamic responses. With the help of GLM, the evoked brain activity could be robustly separated from systemic physiological interference using independent measures of nuisance regressors, such as the short-separation fNIRS measurements (pre-switch *vs*. post-switch) in this study. A recent study has examined the effectiveness of the GLM-based approach and found that it could provide better estimates of brain activity and channel-specific hemodynamic response function regressor (von Lühmann et al., 2020). In this study, the change in HbO (*Y*_Δ*HbO*_) for each channel was estimated based on *time* in the pre-switch or post-switch periods, using R (*Y*_Δ*HbO*_ = *aX time* + *b* +). In particular, the paired results of the pre-switch and post-switch periods for each channel were analyzed to identify the “V-shape curve.” Also, the results indicating the neural correlates of cognitive shifting in different groups and stages are shown in Tables 1–7.

**Table 1:** Comparison of increases in HbO between the Switch (N1 = 25) and Perseverate (N2 = 20) groups in the DCCS task (pre-switch).

	**Group**	**Mean**	**SD**	***T*-Value**	***p*-value**
Channel 1	Switch	−0.040	2.447	1.085	0.284
	Perseverate	−0.746	1.922		
Channel 2	Switch	−0.544	2.902	0.101	0.920
	Perseverate	−0.625	2.463		
Channel 3	Switch	0.583	2.110	0.446	0.661
	Perseverate	0.291	2.268		
Channel 4	Switch	−0.794	2.373	−0.161	0.873
	Perseverate	−0.696	1.703		
Channel 5	Switch	0.309	2.469	−0.349	0.729
	Perseverate	0.621	3.516		
Channel 6	Switch	0.411	2.460	0.879	0.984
	Perseverate	−0.248	2.552		
Channel 7	Switch	−0.021	2.407	0.371	0.712
	Perseverate	−0.286	2.349		
Channel 8	Switch	0.982	2.184	2.210	0.033*
	Perseverate	−0.289	1.677		
Channel 9	Switch	−0.918	2.086	−0.812	0.421
	Perseverate	−0.375	2.396		
Channel 10	Switch	−0.814	2.563	0.186	0.857
	Perseverate	−0.940	1.974		
Channel 11	Switch	−2.139	3.645	−1.750	0.088
	Perseverate	−0.661	1.909		
Channel 12	Switch	−1.789	2.269	0.070	0.944
	Perseverate	−1.837	2.317		
Channel 13	Switch	−2.365	2.489	−1.377	0.176
	Perseverate	−1.313	2.618		
Channel 14	Switch	−1.333	2.256	−1.038	0.305
	Perseverate	−0.595	2.506		
Channel 15	Switch	−1.268	2.361	−1.645	0.107
	Perseverate	−0.092	2.409		
Channel 16	Switch	−1.120	2.123	−0.714	0.479
	Perseverate	−0.644	2.334		
Channel 17	Switch	−2.568	2.406	−0.169	0.866
	Perseverate	−2.443	2.524		

*p < 0.05.

## Results

### Behavioral Results

Altogether, 49 participants completed the DCCS task in this study. Approximately 25 children (M_*age*_ = 68.66, SD = 5.3), including one 4-year-olds, 13 5-year-olds, and 11 6-year-olds, passed all the testing items thus were classified into the Switch group (N_*Switch*_ =25, M_*DCCS*_ = 36, *SD* = 0). Twenty children (M_*age*_ = 62.05, SD = 8.13) made more than one perseverative errors, including nine 4-year-olds, six 5-year-olds, and five 6-year-olds, thus were included in the Perseverate group (N_*Perseverate*_ = 20, M_*DCCS*_ = 28.2, *SD* = 6.83, ranged between 18 and 33). Another four children who only committed one minor mistake were regarded as the marginal case and excluded from the final analysis. No significant age difference was found; thus, 45 children were included in the data analysis (N_*total*_ = 45, M_*DCCS*_ = 32.53, *SD* = 5.96).

### Functional Near-Infrared Spectroscopy Results

#### T-Tests Results

Three sets of *t*-tests were conducted to examine the significant differences in HbO increases between the Switch and Perseverate groups in the 17 channels.

First, a set of independent-sample *t*-tests was conducted to determine whether there were significant differences in the 17 channels between the Switch and Perseverate groups. As multiple channels were involved in this type of *t*-tests, all the results were corrected for multiple comparisons using the false discovery rate (FDR), and the adjusted significance level of *p*-value was set at 0.05. As shown in Table 1, the results indicated a significant between-group difference during the pre-switch period in BA 44 (channel 8) (*t* = 2.21, *p* < 0.05), with the Switch group (M = 0.98, SD = 2.18) having significantly more increase than the Perseverate group (M = −0.29, SD = 1.67). However, there were no significant between-group differences in the other channels in this period. In the post-switch period, as shown in Table 2, the results indicated a significant between-group difference in BA 9 (channel 11) (*t* = −2.16, *p* < 0.05) and BA 10 (ch 15) (*t* = −2.32, *p* < 0.05), with the Switch group significantly less activated than the Perseverate group in both channels. No significant between-group differences were found in the other channels. All these results jointly indicated that (1) in the pre-switch period, the Switch group had significantly more activation in BA 44 than the Perseverate group; (2) in the post-switch period, the Perseverate group had significantly more activation in BA 9 and BA 10 than the Switch group.

**Table 2:** Comparison of increases in HbO between the Switch (N1 = 25) and Perseverate (N2 = 20) groups in the DCCS task (post-switch).

	**Group**	**Mean**	**SD**	***T*-value**	***P*-value**
Channel 1	Switch	−0.876	1.911	−0.240	0.811
	Perseverate	−0.751	1.569		
Channel 2	Switch	−1.450	3.127	−0.1022	0.310
	Perseverate	−0.471	3.246		
Channel 3	Switch	0.068	2.845	−1.148	0.257
	Perseverate	0.923	2.148		
Channel 4	Switch	−1.309	1.853	−1.070	0.291
	Perseverate	−0.695	1.990		
Channel 5	Switch	−0.458	3.484	−0.357	0.723
	Perseverate	−0.077	3.665		
Channel 6	Switch	−0.269	3.045	0.721	0.475
	Perseverate	−0.838	2.243		
Channel 7	Switch	−0.976	2.592	−0.260	0.796
	Perseverate	−0.769	2.728		
Channel 8	Switch	0.186	2.537	0.920	0.363
	Perseverate	−0.382	1.577		
Channel 9	Switch	−1.527	2.528	−1.134	0.263
	Perseverate	−0.699	2.352		
Channel 10	Switch	−1.532	3.132	0.516	0.609
	Perseverate	−2.018	3.146		
Channel 11	Switch	−2.409	4.347	−2.163	0.038*
	Perseverate	−0.310	1.929		
Channel 12	Switch	−2.158	2.818	−0.261	0.796
	Perseverate	−1.965	2.127		
Channel 13	Switch	−2.235	2.554	−0.546	0.588
	Perseverate	−1.843	2.260		
Channel 14	Switch	−1.978	3.160	−1.462	0.151
	Perseverate	−0.711	2.647		
Channel 15	Switch	−2.059	2.026	−2.323	0.025*
	Perseverate	−0.592	2.196		
Channel 16	Switch	−1.640	2.379	−0.728	0.470
	Perseverate	−1.159	2.047		
Channel 17	Switch	−2.710	2.513	0.031	0.975
	Perseverate	−2.732	2.067		

*p < 0.05.

Next, a set of paired-samples *t*-tests was conducted to determine whether there were significant within-group differences in HbO in the Switch group. No significant differences were found between the pre-switch and post-switch periods in all the channels, *t*s < 2.83, *p*s > 0.10. However, as shown in Table 3, significant differences were found between the rest and pre-switch periods in BA 9 (channels 11, 12, 14, and 16) and BA 10 (channels 13, 15, and 17), *t*s > 2.65, *p*s < 0.04, after corrected with FDR. In addition, as shown in Table 4, significant differences were also found between the rest and post-switch periods in BA 6 (channels 1 and 9), BA 9 (channels 11, 12, 14, and 16), BA 10 (channels 13, 15, and 17), BA 40 (channel 4), and BA 44 (channel 2), *t*s > 2.31, *p*s < 0.05, after corrected with FDR.

**Table 3:** Comparison of increases in HbO of the Switch group between the rest and pre-switch in the DCCS task.

**Rest–pre-switch**	**Paired differences**		***T*-Value**	***p*-value**
	**Mean**	**Std. Deviation**		
Channel 1	0.154	2.635	0.292	0.821
Channel 2	0.465	2.944	0.791	0.495
Channel 3	−0.658	2.198	−1.497	0.227
Channel 4	0.930	2.576	1.806	0.141
Channel 5	−0.427	2.406	−0.889	0.465
Channel 6	−0.584	2.308	−1.266	0.285
Channel 7	0.009	2.501	0.019	0.985
Channel 8	−1.048	2.172	−2.412	0.051
Channel 9	0.916	2.062	2.222	0.068
Channel 10	0.683	2.702	1.265	0.285
Channel 11	2.279	3.685	3.092	0.021*
Channel 12	1.985	2.422	4.098	0.000
Channel 13	2.335	2.719	4.293	0.000
Channel 14	1.300	2.250	2.889	0.025*
Channel 15	1.309	2.465	2.656	0.034*
Channel 16	1.250	2.188	2.857	0.025*
Channel 17	2.448	2.530	4.838	0.000

*p < 0.05.

**Table 4:** Comparison of increases in HbO of the Switch group between the rest and post-switch in the DCCS task.

**Rest–post-switch**	**Paired differences**		***T*-Value**	***p*-value**
	**Mean**	**Std. Deviation**		
Channel 1	0.990	1.993	2.484	0.034*
Channel 2	1.372	2.962	2.316	0.045*
Channel 3	−0.143	2.838	−0.252	0.853
Channel 4	1.446	2.072	3.488	0.006**
Channel 5	0.341	3.513	0.485	0.716
Channel 6	0.096	2.889	0.166	0.87
Channel 7	0.964	2.616	1.842	0.102
Channel 8	−0.252	2.512	−0.502	0.7163
Channel 9	1.526	2.425	3.146	0.010**
Channel 10	1.402	3.222	2.176	0.056
Channel 11	2.550	4.233	3.012	0.011*
Channel 12	2.354	2.889	4.075	0.000***
Channel 13	2.206	2.653	4.157	0.000***
Channel 14	1.945	3.115	3.121	0.011*
Channel 15	2.100	2.098	5.005	0.000***
Channel 16	1.771	2.410	3.673	0.003**
Channel 17	2.591	2.646	4.895	0.000***

*p < 0.05; **p < 0.01; ***p < 0.001.

Third, a set of paired-samples *t*-tests was conducted to determine whether there were significant within-group differences in HbO in the Perseverate group. No significant differences were found between the pre-switch and post-switch periods in all the channels, *t*s < 2.37, *p*s > 0.49. However, as shown in Table 5, significant differences were found between the rest and pre-switch periods in BA 9 (channel 12) and BA 10 (channel 17), *t*s > 3.28, *p*s < 0.04, after corrected with FDR. In addition, as shown Table 6, significant differences were found between the rest and post-switch periods in BA 8 (channel 10), BA 9 (channel 12), and BA 10 (channels 13 and 17), *t*s > 2.83, *p*s < 0.05, after corrected with FDR.

**Table 5:** Comparison of increases in HbO of the Perseverate group between the rest and pre-switch in the DCCS task.

**Rest–pre-switch**	**Paired differences**		***T*-value**	***p*-value**
	**Mean**	**Std. Deviation**		
Channel 1	0.552	2.045	1.208	0.460
Channel 2	0.366	2.900	0.564	0.710
Channel 3	−0.252	2.997	−0.376	0.805
Channel 4	0.636	1.655	1.719	0.356
Channel 5	−0.434	3.458	−0.561	0.710
Channel 6	0.315	2.533	0.556	0.710
Channel 7	0.125	2.593	0.216	0.865
Channel 8	0.301	1.734	0.776	0.710
Channel 9	0.381	2.371	0.719	0.700
Channel 10	0.946	1.912	2.212	0.165
Channel 11	0.699	2.202	1.420	0.460
Channel 12	1.703	2.319	3.284	0.034*
Channel 13	1.392	2.578	2.415	0.147
Channel 14	0.645	2.399	1.202	0.460
Channel 15	0.088	2.281	0.172	0.865
Channel 16	0.653	2.278	1.282	0.460
Channel 17	2.286	2.560	3.993	0.017*

*p < 0.05.

**Table 6:** Comparison of increases in HbO of the Perseverate group between the rest and post-switch in the DCCS task.

**Rest–post-switch**	**Paired differences**		***T*-value**	***p*-value**
	**Mean**	**Std. Deviation**		
Channel 1	0.557	1.590	1.568	0.292
Channel 2	0.212	3.319	0.286	0.778
Channel 3	−0.883	2.584	−1.528	0.292
Channel 4	0.635	1.918	1.480	0.292
Channel 5	0.265	3.553	0.334	0.778
Channel 6	0.905	2.285	1.771	0.263
Channel 7	0.608	2.644	1.028	0.384
Channel 8	0.393	1.398	1.257	0.304
Channel 9	0.706	2.401	1.314	0.304
Channel 10	2.024	3.189	2.839	0.042*
Channel 11	0.349	2.079	0.750	0.523
Channel 12	1.831	2.110	3.880	0.008**
Channel 13	1.922	2.555	3.364	0.017*
Channel 14	0.761	2.566	1.327	0.304
Channel 15	0.588	2.134	1.233	0.304
Channel 16	1.168	1.961	2.662	0.051
Channel 17	2.575	2.057	5.598	0.000***

*p < 0.05; **p < 0.01; ***p < 0.001.

#### General Linear Model Results

Two sets of GLM analyses were conducted to model the changes in HbO for the Switch and Perseverate groups to test the V-shape curve hypothesis.

**Figure 5:**
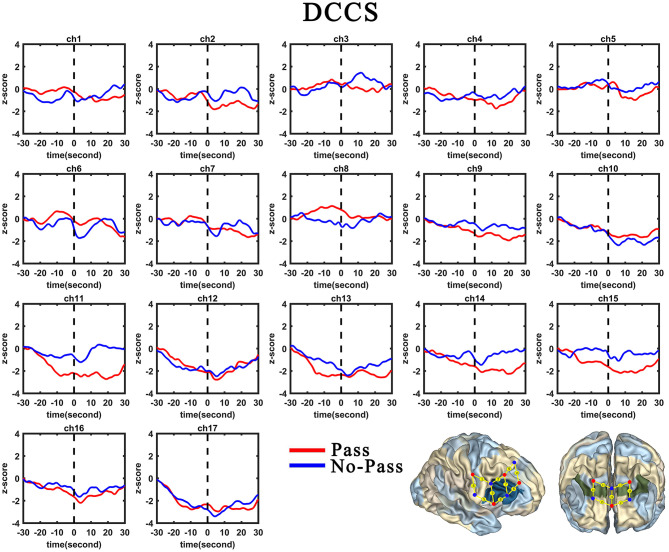
Temporal changes in the HbO concentration in the 17 channels during the DCCS task. From left to right are channels 1–17. HbO data for the Switch and Perseverate group are shown in red and blue line, respectively.

First, the GLM results for the Switch group (N_*switch*_ = 25) are presented in Table 7 and Figure 5. During the pre-switch period, significant HbO decreases were observed in BA 6 (channel 9), BA 8 (channel 10), BA 9 (channels 11, 12, 14, and 16), BA 10 (channels 13 and 15), BA 40 (channel 4), and BA 44 (channels 2, 5, 6, and 8), *F*s > 4.08 (for the models), *t*s < −2.02 (for β), *p*s < 0.05. In contrast, remarkable increases in HbO were found in BA 6 (channel 1) and BA 44 (channels 3 and 7), *F*s > 10.96 (for the models), *t*s > 3.31 (for β), *p*s < 0.01. During the post-switch period, significant HbO decreases were observed in BA 6 (channels 1 and 9), BA 9 (channels 11 and 14), BA 10 (channels 13, 15, and 17), BA 40 (channel 4), and BA 44 (channels 3, 5, 6, and 7), *F*s > 8.86 (for the models), *t*s < −2.97 (for β), *p*s < 0.01. In contrast, remarkable increases in HbO were found in BA 9 (channels 12 and 16) and BA 44 (channel 2), *F*s > 39.78 (for the models), *t*s > 6.30 (for β), *p*s <0.001. Accordingly, the V-shaped curve was found in the GLM results for BA 9 (channels 12 and 16) and BA 44 (channel 2), indicating that these areas were highly associated with the cognitive shifting in the Switch group.

**Table 7:** Predicting increase in HbO for the Switch group (N1 = 25) in the DCCS Task.

**Channel**	**Pre-switch**				**Post-switch**				**Observed shifting**
	**β**	**Δ*R*^**2**^**	***F*-value**	***T*-value**	**β**	**Δ*R*^**2**^**	***F*-value**	***T-*value**	
Ch 1	0.689	0.470	88.792***	9.723***	−0.790	0.621	163.153***	−12.773***	
Ch 2	−0.566	0.313	46.131***	−6.792***	0.537	0.281	39.782***	6.307***	Yes
Ch 3	0.774	0.595	146.516***	12.104***	−0.821	0.671	202.521***	−14.231***	
Ch 4	−0.851	0.721	256.416***	−16.013***	−0.736	0.537	115.874***	−10.765***	
Ch 5	−0.200	0.030	4.089*	−2.022*	−0.840	0.703	235.097***	−15.333***	
Ch 6	−0.613	0.369	58.979***	−7.680***	−0.510	0.253	34.503***	−5.874***	
Ch 7	0.317	0.091	10.966**	3.311**	−0.810	0.656	186.914***	−13.672***	
Ch 8	−0.781	0.606	153.517***	−12.390***	−0.053	−0.007	0.277	−0.526	
Ch 9	−0.927	0.858	598.679***	−24.468***	−0.760	0.573	133.650***	−11.561***	
Ch 10	−0.920	0.844	538.001***	−23.195***	0.085	−0.003	0.719	0.848	
Ch 11	−0.723	0.519	107.622***	−10.374***	−0.477	0.220	28.879***	−5.374***	
Ch 12	−0.946	0.894	834.446***	−28.887***	0.916	0.837	510.042***	22.584***	Yes
Ch 13	−0.436	0.181	22.951***	−4.791***	−0.439	0.185	23.449***	−4.842***	
Ch 14	−0.942	0.886	769.913***	−27.747***	−0.505	0.248	33.571***	−5.794***	
Ch 15	−0.703	0.489	95.568***	−9.776***	−0.433	0.179	22.599***	−4.754***	
Ch 16	−0.886	0.783	359.173***	−18.952***	0.867	0.749	295.914***	17.202***	Yes
Ch 17	−0.161	0.016	2.594	−1.611	−0.288	0.074	8.865**	−2.977**	

*p < 0.05; **p < 0.01; ***p < 0.001.

Second, the GLM results for the Perseverate group (N1 = 25) are presented in Table 8 and Figure 5. During the pre-switch period, significant HbO decreases were observed in BA 6 (channel 9), BA 8 (channel 10), BA 9 (channels 11, 12, 14, and 16), BA 10 (channels 13, 15, and 17), and BA 44 (channels 6 and 8), *F*s > 11.67 (for the models), *t*s < −3.41 (for β), *p*s < 0.01. In contrast, remarkable increases in HbO were found in BA 6 (channel 1), BA 40 (channel 4), and BA 44 (channels 2, 3, and 5), *F*s > 11.29 (for the models), *t*s > 3.36 (for β), *p*s < 0.01. During the post-switch period, significant HbO decreases were observed in BA 8 (channel 10) and BA 44 (channel 5), *F*s > 5.30 (for the models), *t*s < −2.30 (for β), *p*s < 0.05. In contrast, remarkable increases in HbO were found in BA 6 (channel 1), BA 9 (channels 11, 12, 14, and 16), BA 10 (channels 13, 15, and 17), and BA 44 (channels 2, 3, 6, 7, and 8), *F*s > 49.30 (for the models), *t*s > 7.02 (for β), *p*s < 0.001. Accordingly, the V-shaped curve was found in the GLM results for BA 9 (channels 11, 12, 14, and 16), BA 10 (channels 13, 15, and 17), and BA 44 (channels 6 and 8), indicating that these areas were highly associated with the cognitive shifting in the Perseverate group.

**Table 8:** Predicting increase in HbO for the Perseverate group (N2 = 20) in the DCCS task.

**Channel**	**Pre-switch**				**Post-switch**				**Observed shifting**
	**β**	**Δ*R*^**2**^**	***F*-value**	***T-*value**	**β**	**Δ*R*^**2**^**	***F*-value**	***T-*value**	
Ch 1	0.833	0.691	222.828***	14.927***	0.839	0.700	232.500***	15.248***	
Ch 2	0.321	0.094	11.294**	3.361**	0.767	0.585	140.445***	11.851***	
Ch 3	0.869	0.753	302.640***	17.739***	0.593	0.345	53.127***	7.289***	
Ch 4	0.887	0.785	362.922***	19.051***	−0.122	0.005	1.492	−1.221	
Ch 5	0.895	0.801	395.037***	19.876***	−0.227	0.042	5.308*	−2.304*	
Ch 6	−0.326	0.097	11.677**	−3.417**	0.933	0.869	657.467***	25.641***	Yes
Ch 7	0.131	0.007	1.708	1.307	0.753	0.562	128.171***	11.321***	
Ch 8	−0.875	0.763	320.568***	−17.904***	0.898	0.804	406.019***	20.150***	Yes
Ch 9	−0.387	0.141	17.268***	−4.156***	−0.050	−0.008	0.249	−0.499	
Ch 10	−0.927	0.857	595.749***	−24.408***	−0.268	0.063	7.606**	−2.758**	
Ch 11	−0.453	0.197	25.299***	−5.030***	0.770	0.588	142.570***	11.940***	Yes
Ch 12	−0.606	0.361	56.866***	−7.541***	0.834	0.693	224.230***	14.974***	Yes
Ch 13	−0.962	0.924	1210.151***	−34.787***	0.828	0.683	213.912***	14.626***	Yes
Ch 14	−0.374	0.131	15.943***	−3.993***	0.878	0.768	328.698***	18.130***	Yes
Ch 15	−0.328	0.098	11.812**	−3.437**	0.579	0.328	49.308***	7.022***	Yes
Ch 16	−0.868	0.751	299.845***	−17.316***	0.637	0.399	66.763***	8.171***	Yes
Ch 17	−0.925	0.854	581.968***	−24.124***	0.825	0.678	209.644***	14.479***	Yes

*p < 0.05; **p < 0.01; ***p < 0.001.

## Discussion

### Two Patterns of Neural Correlates of Cognitive Shifting

This study found two different patterns of neural correlates of cognitive shifting. The Switch Pattern: in the pre-switch period, the children only activated BA 9 and BA 10 to complete the card-sorting task, whereas in the post-switch period, they activated BA 6, BA 9, BA 10, BA 40, and BA 44. The Perseverate Pattern: in the pre-switch period, the children only activated BA 9 and BA 10, whereas in the post-switch period, they activated BA 8, BA 9, and BA 10. There were no significant differences between pre-switch and post-switch periods within the two patterns. This finding indicates that the paired *t*-tests, which have been extensively used in neuroimaging studies, cannot identify the differences between the pre- and post-switch periods. Therefore, some more appropriate statistical analyses such as GLM are needed in this study. Nevertheless, the two patterns differed in the post-switch period: the Switch group activated BA 6, BA 9, BA 10, BA 40, and BA 44, whereas the Perseverate group activated BA 8, BA 9, and BA 10. Furthermore, the between-group comparison found that in the pre-switch period, the Switch group had significantly more activation in BA 44. In the post-switch period, the Perseverate group had significantly more activation in BA 9 and BA 10. All these *t*-tests results jointly indicated that BA 9 and BA 10 were substantially involved in the DCCS task for both Switch and Perseverate groups, and BA 44 might play a critical role in the successful completion of the cognitive shifting. This section will discuss the roles of BA 9, BA 10, BA 40, and BA44 in the DCCS tasks.

First, the existing studies have indicated that BA 9 is involved in attributing attention, the theory of mind, working memory, spatial memory, recognition, recall, and planning (Gallagher et al., 2002; Leung et al., 2002; Pochon et al., 2002; Raye et al., 2002; Zhang et al., 2003; Slotnick and Moo, 2006). The cognitive shifting task in this study calls for the engagement of working memory, spatial memory, recognition, recall, and planning, thus involving both hemispheres of BA 9. In addition, completing the DCCS task involves a set of neural correlates such as BA 9 (channel 12), BA 10 (channel 17), BA 40 (channel 4), and BA 44 (channel 3/5/8), which are working as a team to jointly and collaboratively complete the different cognitive jobs. According to the DNF model (Perone et al., 2015, 2019), the substantial engagement of both hemispheres of BA 9 is critical to completing the DCCS task, as it is the neural correlate of attention, working memory, conflict monitoring, and problem-solving. Therefore, in both pre-switch and post-switch periods, the Switch children have fully activated all the channels of BA 9 (see Table 9), which might be in charge of different EF functions such as storing, alerting, monitoring, and controlling (Wu et al., 2020). However, those Perseverate children might not be able to activate all the channels of BA 9 to achieve its full functioning and, accordingly, failed to complete the task. This *t*-tests result has been verified by the GLM results and will be discussed in the next section.

**Table 9:** Observed significant differences in HbO by fNIRS.

**Group**	**Channel**																
	**1**	**2**	**3**	**4**	**5**	**6**	**7**	**8**	**9**	**10**	**11**	**12**	**13**	**14**	**15**	**16**	**17**
Pre-switch: Switch *vs*. Perseverate								X									
Post-switch: Switch *vs*. Perseverate											X				X		
Switch: rest vs. pre-switch											X	X	X	X	X	X	X
Switch: rest vs. post-switch	X	X		X					X		X	X	X	X	X	X	X
Switch: pre- vs. post-switch																	
Perseverate: rest vs. pre-switch												X					X
Perseverate: rest vs. post-switch										X		X	X				X
Perseverate: pre- vs. post-switch																	
Switch (V-shape)		X										X				X	
Perseverate (V-shape)						X		X			X	X	X	X	X	X	X
Brodmann Area (BA)	6	44	44	40	44	44	44	44	6	8	9	9	10	9	10	9	10

Next, BA 10 has been involved in cognitive shifting for the Switch and Perseverate children. As part of the pre-frontal cortex, BA 10 is engaged in strategic processes in memory recall and various executive functions. Hyafil and Koechlin (2016) have proposed that the processing of “cognitive branching” is the core function of BA 10. Cognitive branching enables a previously running task to be maintained in a pending state for subsequent retrieval and execution upon completion of the ongoing one, which is decisive to rule shifting in the DCCS task. In this study, the Switch group has activated all the channels in BA 10 during both pre- and post-switch periods, whereas the Perseverate group failed to do so. All these findings jointly demonstrate the importance of involving BA 10 in the DCCS cognitive shifting. This finding, however, will be verified with the GLM results in the next section.

Third, significant activation in BA 40 was found in the Switch group. BA 40 is located in the temporoparietal junction and involved in semantic processing, working memory, executive control of behavior, motor planning, and response to visual motion. The two functional magnetic resonance imaging studies by Morton et al. (2009) and Ezekiel et al. (2013) found that BA 40 was significantly activated during the DCCS task in school-aged children. This study found that BA 40 was also involved in the Switch children's cognitive shifting, ranging from age 4 to 6 years. Those Perseverate children, however, did not show significant activation of BA 40. All these results jointly demonstrate that the temporoparietal junction area also plays an important role in the cognitive shifting of the DCCS task. Nevertheless, this finding will be further examined with the GLM results in the next section.

Last, this study found the significant activation of BA 44 in the Switch group during the DCCS task. BA 44 functions significantly in binding the language elements, selecting information among competing sources, generating/extracting action meanings, and cognitive control mechanisms for the syntactic processing of sentences (Aron et al., 2004). Besides, BA 44 is responsible for cognitive shifting in the DCCS task in preschool children and hand movements (Moriguchi and Hiraki, 2009). In the DCCS study, children should make appropriate hand movements to return the cards to the correct box; thus, hand movement is substantially involved and could be regarded as critical to completing the cognitive shifting. Therefore, one might challenge the real role of BA 44 in this task: is it for cognitive shifting or hand movements? This study can confirm its function in cognitive shifting for two reasons. First, all the participants in this study were right-handed; thus, their hand movement would not engage the right-hemisphere BA 44, which is exactly in charge of their left-hand movements. Second, we will use the V-shape curve model to test whether BA 44 is responsible for cognitive shifting or hand movement in the following section.

### V-Shape Curve Hypothesis

The GLM results identified significant HbO decreases in BA 6, BA 8 (pre-switch only), BA 9, BA 10, BA 40, and BA 44 for the Switch group during the pre-switch and post-switch periods. Similarly, the GLM results also confirmed significant HbO decreases in BA 6, BA 8, BA 9, BA 10, and BA 44 during the pre-switch period and in BA 8 and BA 44 during the post-switch period for the Perseverate group. The significant decrease in HbO in these brain areas indicated that there might be a kind of habituation during the DCCS task: when the same card-sorting tasks were repeated over and over, there were reduced responses from the same neural correlates and, accordingly, a decrease in HbO in the blood (Purves et al., 2018). Other theories and hypotheses, however, might not be able to explain why there were significant decreases in these neural correlates during the DCCS task.

Meanwhile, the GLM results have also confirmed the significant increase in HbO during the pre-switch period: BA 6 and BA 44 for the Switch group and BA 6, BA 40, and BA 44 for the Perseverate group. In addition, during the post-switch period, the Switch group had a significant increase in BA 9 and BA 44. In contrast, the Perseverate group had a significant increase in BA 6, BA 9, BA 10, and BA 44. All these results jointly indicated that BA 6 and BA 44 were commonly activated during the pre-switch period, and BA 9 and BA 44 were commonly activated during the post-switch period for both Switch and Perseverate groups.

However, the GLM results discussed earlier could not confirm whether those BAs with a significant increase and/or decrease in HbO were really in charge of the cognitive shifting of the DCCS task. According to our “V-shape curve” hypothesis, only those brain areas with a significant decrease in the pre-switch period and a significant increase in the post-switch period could be regarded as the neural correlations of cognitive shifting. This is because the paired decrease (pre-switch) and increase (post-switch) in HbO do reflect a process of habituation-plus-dishabituation (Purves et al., 2018), which is similar to the HaND paradigm (Lloyd-Fox et al., 2019). In this study, the GLM analysis results have provided empirical evidence to support this “V-shape curve” hypothesis by identifying a significant habituation-plus-dishabituation cycle in BA 9 and BA 44 for the Switch group and BA 9, BA 10, and BA 44 for the Perseverate group. This finding has further verified the results of the *t*-test and jointly demonstrate that BA 9 and BA 44 are the real neural correlations of cognitive shifting, with BA 10 playing a compensatory role for the Perseverate group.

In the past decade, the role of BA 10 has been poorly understood. A recent meta-analysis has found that it is involved in working memory, episodic memory, and multiple-task coordination (Gilbert et al., 2006). In the cognitive shifting during the DCCS task, those Perseverate children had to activate BA 10 to help BA 9 and BA 44 complete the complicated cognitive tasks. This could be explained with neural compensatory mechanisms recently found by Yoon et al. (2019). In their fNIRS study of the normal elderly group and amnestic and non-amnestic mild cognitive impairment groups, significantly higher activation of the right pre-frontal cortex was found as the compensatory effect to supplant left pre-frontal function in the Stroop tests. Although the neuroplasticity of the right pre-frontal cortex has been reported in other studies (Yoon et al., 2019), this is the first study to identify the neural compensatory mechanism of the right pre-frontal cortex in the cognitive shifting tasks in young children. Further studies with younger children are needed to explore the origin of this neural compensatory mechanism.

Last but not least, the GLM analysis did not find any significant habituation-plus-dishabituation cycle in BA 40. This GLM finding is not consistent with the result of the *t*-test. However, this discrepancy implies that BA 40 might be involved in the DCCS tasks (judged by *t*-tests) but might not necessarily be responsible for the cognitive shifting (examined by GLM). Nevertheless, future studies should be conducted to further explore the differences between *t*-tests and GLM results.

## Conclusions, Limitations, and Implications

This study has provided fNIRS evidence to support the two hypotheses. First, two patterns of neural correlates of the DCCS task were found: (1) the Switch Pattern: BA 9 and BA 10 were activated in the pre-switch period, whereas BA 6, BA 9, BA 10, BA 40, and BA 44 were activated in the post-switch period; (2) the Perseverate Pattern: BA 9 and BA 10 were activated in the pre-switch period, whereas BA 8, BA 9, and BA 10 were activated in the post-switch period. Both BA 9 and BA 10 were commonly involved in the DCCS task by the Switch and Perseverate children. In addition, BA 44 plays a critical role in successful cognitive shifting. Second, this study has confirmed the “V-shape curve” hypothesis by identifying a significant decrease–increase cycle in BA 9 and BA 44, the neural correlates of the cognitive shifting. Also, BA 10 plays a compensatory role for the Perseverate group to complete the cognitive shifting. In summary, BA 6, BA 8, BA 9, BA 10, BA 40, and BA 44 were generally involved thus could be regarded as the general neural network of processing the DCCS task in preschoolers. However, only BA 9 and BA 44 were found with the specific neural correlates of the cognitive shifting during the DCCS task. In the Perseverate group, BA 10 was also activated to compensate for BA 9 and BA 44.

However, this study has some limitations. First, the procedure was slightly different from those initially designed by Moriguchi and Hiraki (2009). This study asked the young children to complete three rounds of cognitive shifting, presented with 36 cards of color and shape variations in a fixed sequence. This design was based on the habituation–dishabituation paradigm, which could help identify the decrease–increase cycle in HbO. In the future, a mixed arrangement should be adopted to increase the difficulty. Second, other brain regions might also contribute to the development of cognitive shifting. However, with a very limited number of channels, this study could only focus on the right inferior frontal cortex and right and left pre-frontal areas. More channels should be used to explore other brain areas' possible activation, using various tasks and even longitudinal data in the future. Third, the habituation rate might have an individual difference. In this study, we simply classified the participating children by their DCCS performance rather than by their age, as we believe that the individual difference in brain maturation might be greater than the age difference. Accordingly, we believe that Switch children tend to have the same habituation rate. However, this might not be true and needs more empirical evidence. Nevertheless, this study, for the first time, has proposed and confirmed a new hypothesis—the V-shaped curve in regression lines—to identify the occurrence of cognitive shifting in the DCCS task. This method will provide a reliable and direct indicator of cognitive shifting, which could be used to explore younger children's performance in the DCCS task.

## Data Availability Statement

The raw data supporting the conclusions of this article will be made available by the authors, without undue reservation.

## Ethics Statement

The studies involving human participants were reviewed and approved by Ethics Committee, Faculty of Medicine, Shenzhen University. Written informed consent to participate in this study was provided by the participants' legal guardian/next of kin.

## Author Contributions

DW, JY, SX, and JL collected the data. DW and HL designed the experiment. JY and CC analyzed the data. DW, JY, CC, and HL drafted the manuscript. All authors contributed to the article and approved the submitted version.

## Conflict of Interest

The authors declare that the research was conducted in the absence of any commercial or financial relationships that could be construed as a potential conflict of interest.
